# Mandatory Naptimes in Childcare do not Reduce Children’s Cortisol Levels

**DOI:** 10.1038/s41598-018-22555-8

**Published:** 2018-03-14

**Authors:** Karen J. Thorpe, Cassandra L. Pattinson, Simon S. Smith, Sally L. Staton

**Affiliations:** Institute for Social Science Research (ISSR), The University of Queensland, Long Pocket Precinct, 80 Meiers Rd, Indooroopilly, Queensland 4068 Australia

## Abstract

The majority of preschool children (aged 3–5 years) no longer habitually nap, yet in childcare settings daily mandated naptimes in which children lie down without alternative activity remains a common practice. Mandated naptimes are associated with observed reductions in emotional climate and increased incidence of distress. While intended to be restful, mandatory naptimes may induce stress in those children unable to sleep. To examine this possibility, we applied a 2 (mandated/flexible practice) × 2 (nap/no-nap) design to test group difference in stress responses of children (*N* = 43, mean age 56.3 months). Salivary cortisol level was measured at 4 time-points (waking, pre-naptime, post-naptime, and bedtime) across two days at childcare. Overall our results show a significant decline in cortisol level from wake to pre-naptime and from post-naptime to bedtime. No significant change in cortisol level was observed from pre- to post- naptime. Significant group differences in cortisol patterns were observed. Notably, children under mandatory naptime conditions who did not nap showed no significant reduction in cortisol level from post-naptime to bedtime. While cortisol measurement suggests naptime is neither stressful nor restful for children in any group, implications for bedtime arousal are raised for those unable to sleep under conditions of mandated naptimes.

## Introduction

Early childhood is a period of normative transition in sleep distribution, during which sleep consolidates into the night as daytime sleep commensurately ceases^1^. Large population studies across a range of nations indicate that, by age 4, fewer than a third of children habitually nap^2,3^. Yet in childcare settings it remains common practice to schedule a naptime for all children through to school entry at age 5 years, regardless of their needs or preferences. While some childcare centres are flexible in their approach, allowing non-nappers to engage in alternative activities (e.g. looking at books), many mandate a naptime for all children, often of a prolonged duration, during which no alternative activities are permitted^4–7^. While the aim of this practice is to promote sleep and provide opportunity for rest during the childcare day, for those unable to sleep, naptime may in fact be stressful^8^.

Existing evidence from observational studies identifies a general pattern of escalating negative emotion associated with naptime practices in childcare. An Australian study of 130 pre-school rooms, observed systematically across the day, found that classroom emotional negativity escalated between learning sessions and the scheduled naptime when children were required to lie down even if not sleeping^8^. The level of emotional negativity was related to the duration of mandated naptime, with those of longest duration associated with most interpersonal negativity, greater behavioural management problems, and highest incidence of behavioural distress. The findings of this study may underestimate effects, however, as they are limited to externalising response. To date, internalising responses (e.g. anxiety) in relation to naptime practices have not been documented.

Cortisol, a physiological marker of stress, presents the potential to measure an individual’s emotional response to naptime practices. Typical diurnal patterns of cortisol in early childhood show a peak at 30 minutes after morning waking (the cortisol awakening response; CAR) followed by a general decline across the day^9,10^. This pattern has been observed in home settings, but not always within childcare settings, where a pattern of rising levels of cortisol across the childcare day has been reported^11–13^. This rise has been attributed to the social stress of the childcare environment and child characteristics, such as age and temperament^14^, however, napping and care practices may contribute to the findings. To date only two prior studies have specifically examined the association between naptime in childcare and salivary cortisol levels. Watamura and colleagues^13^ studied naptime effects on diurnal cortisol patterns in a childcare centre with a mandatory naptime of 50 minutes. While finding a rise in cortisol level across the childcare day, they reported no effect due to children’s rest duration or quality. Ward and colleagues^15^ conducted a study of two centres in which 150 minutes of mandated sleep were implemented. They reported that children who slept during this period had lower cortisol levels, while those who did not sleep evidenced behavioural distress and higher cortisol levels post-naptime. Higher afternoon cortisol was associated with anxious behavior. The authors suggested their findings show a benefit of napping for reduction in stress, but did not consider an alternative explanation; mandated naptime for children who cannot sleep may in fact be the source of stress evidenced in higher cortisol levels. Neither study examined full diurnal patterning, restricting cortisol measurement to the childcare day. Yet to fully understand children’s stress response to naptime requires consideration of the “fit” between naptime practice and sleep need, and extension of cortisol measurement to examine the entire day. This was the aim of the current study that included direct observation of childcare practice, objective sleep measurement using actigraphy and diurnal salivary cortisol in a sample of 43 preschool children (age 36 to 52 months; *M* = 56.3 months).

## Results

### Sleep Practices and Napping Behaviours

**Figure 1:**
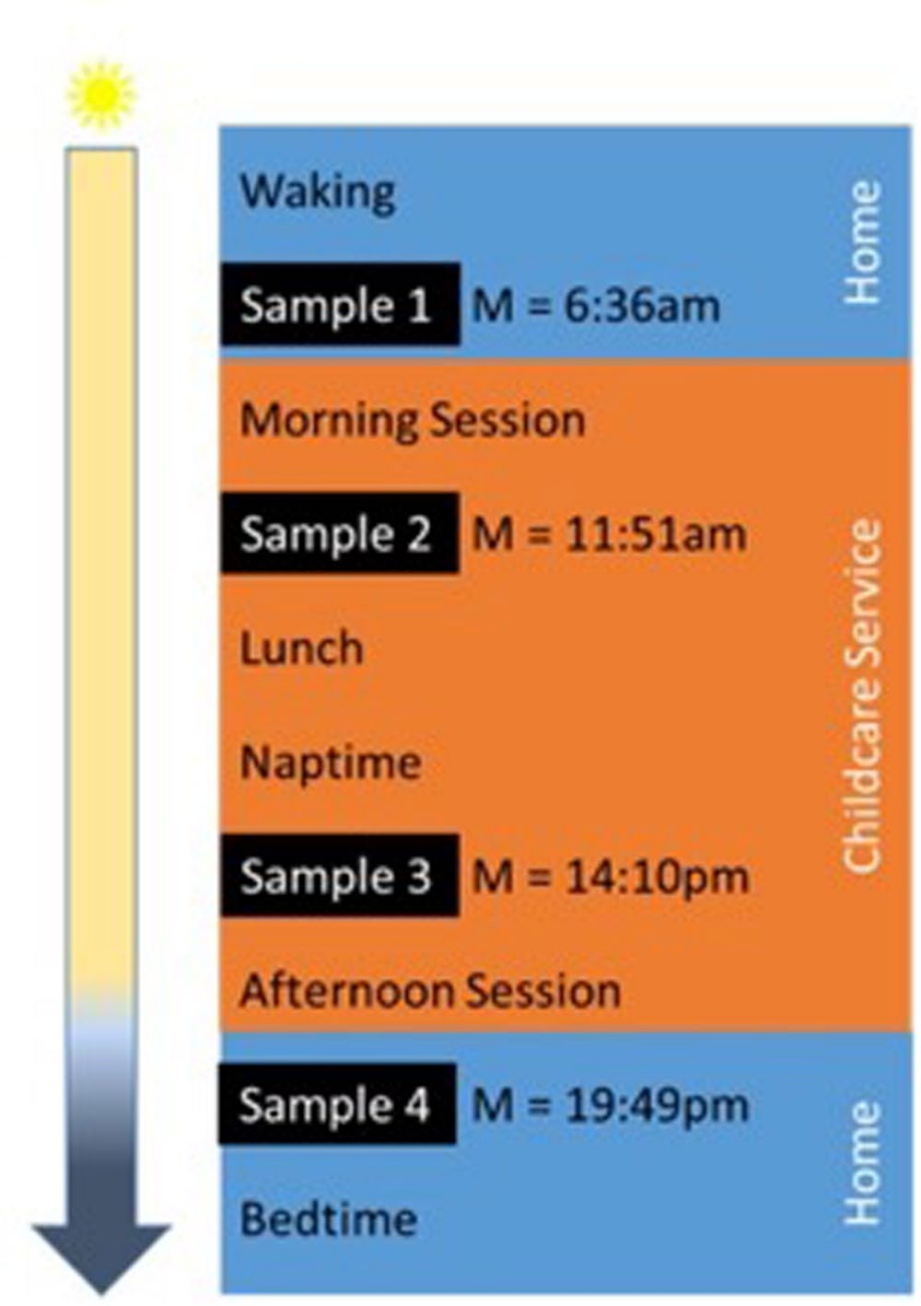
Daily schedule of events and the timing of the samples across the day. The blue boxes represent the time when children are at their homes and the samples were collected by parents (at waking and bedtime). The orange box depicts the order of events at the childcare service with samples collected by the research staff. ‘M’ represents the mean time of cortisol collection for each sample (*N* = 43).

Characteristics of sleep practices across mandatory and flexible rooms are provided in Table 1. Among the study children who napped (*n* = 14), the average duration of napping was 88.9 (*SD* = 38.6) minutes. Average time of cortisol sampling, alongside daily program schedules, is provided in Fig. 1.

**Table 1:** Characteristics of mandatory and flexible room types.

	Mandatory	Flexible
*N* Childcare Rooms	4	2
Average Child:Teacher Ratio	1:9	1:9.5
Scheduled Naptime* [mins]	120	120
Average (SD) Mandatory Naptime^ [mins]	102 (39)	0 (0)
Average (SD) Number of Children Room	20 (3.0)	24 (5.3)
Average (SD) Percentage of Sleeping Children/Room^†^	43.3 (15.9)	1.9 (0.1)

Note. Values averaged across two observation days/room. *Total time allocated for sleep or rest within the daily program. ^Average duration of time in which all children are required to lie on their bed without any alternative activity permitted. ^†^Percentage calculated based on maximum number of children observed eyes closed/lying still during observations.

Children were classified into 3 groups determined by childcare naptime practice (mandatory or flexible) and napping behaviour (nap/no-nap); mandatory naptime no-nap (MNN), mandatory naptime nap (MN), and flexible naptime no-nap (FNN). As napping was uncommon in flexible rooms (*N* = 1 study child on a single day), a balanced design including a flexible nap group was not possible. Sample characteristics across each of the study groups are shown in Table 2.

**Table 2:** Group differences in sample characteristics of mandatory by napping status groups.

	Total	MNN	MN	FNN	P
*N*	43	15	14	14	
Age [months]	56.1 (5.30) [41–64]	55.0 (4.2) [50–63]	54.0 (6.6) [41–62]	59.4 (3.3) [54–64]	**0**.**02**
Female (%)	23 (53.5)	7 (46.7)	9 (65.3)	7 (50.0)	0.61^
**Sampling time**					
Wake	6:36 (0:43) [5:10–8:00]	7:00 (0:38) [5:42–8:00]	6:19 (0:37) [5:10–7:20]	6:27 (0:44) [5:10–7:50]	**0**.**02**^**#**^
Pre-nap	11:51 (0:32) [11:07–12:53]	11:37 (0:19) [11:07–12:03]	11:28 (0:10) [11:17–11:58]	12:30 (0:19) [11:48–12:53]	0.54
Post-nap	14:10 (0:09) [13:44–14:29]	14:08 (0:10) [13:44–14:26]	14:09 (0:10) [13:47–14:29]	14:12 (0:06) [14:04–14:24]	0.46^#^
Bedtime	19:49 (0:51) [18:10–22:40]	19:39 (1:00) [18:10–22:40]	20:13 (0:57) [19:00–22:00]	19:38 (0:25) [18:50–20:17]	0.30
BMI-Z score	0.039 (0.7) [−1.2–1.9]	0.007 (0.6) [−.7–1.2]	0.329 (0.6) [−1.0–1.4]	−0.215 (0.8) [−1.2–1.9]	0.11^#^
Child temperament	50.1 (8.4) [29–61]	47.8 (6.8) [39–58]	47.9 (11.5) [29–60]	54.4 (4.5) [44–61]	0.11
Total Difficulties Score	7.9 (5.7) [0–21]	7.8 (5.9) [2–21]	9.7 (5.6) [1–21]	6.1 (5.5) [0–16]	0.23
Family income	5.2 (2.8) [1–14]	4.3 (1.0) [3–6]	5.5 (3.4) [1–14]	5.7 (3.2) [2–14]	0.48
Parent education	5.6 (1.5) [3–8]	5.85 (1.6) [4–8]	5.7 (1.9) [3–8]	5.4 (1.2) [4–7]	0.71

Note. (SD) [Range]. MNN = mandatory no-nap; MN = mandatory nap; FNN = flexible no-nap. Analyses show non-parametric Independent Samples Kruskall-Wallis test with Mann-Whitney post-hoc tests, unless otherwise specified. ^Pearson’s Chi-Squared Test. ^#^Independent groups ANOVA with Bonferonni adjusted post-hoc tests indicated.

Significant group differences were found for age (χ^2^(2) = 8.11, *p* = 0.02) and time of sampling at wake (*F*[2, 40] = 4.29, *p* = 0.02). Children in the FNN group were significantly older than those within MNN (*p* = 0.04) and MN (*p* = 0.04); whilst children in the MN group had morning wake-times that where significantly earlier than those in the MNN group (*p* = 0.03). No other significant group differences were found. No significant association between these variables and any of the cortisol measures were found (Supplementary Table 1). As such all following analyses are presented unadjusted for covariates.

### Diurnal Cortisol Patterns

**Figure 2:**
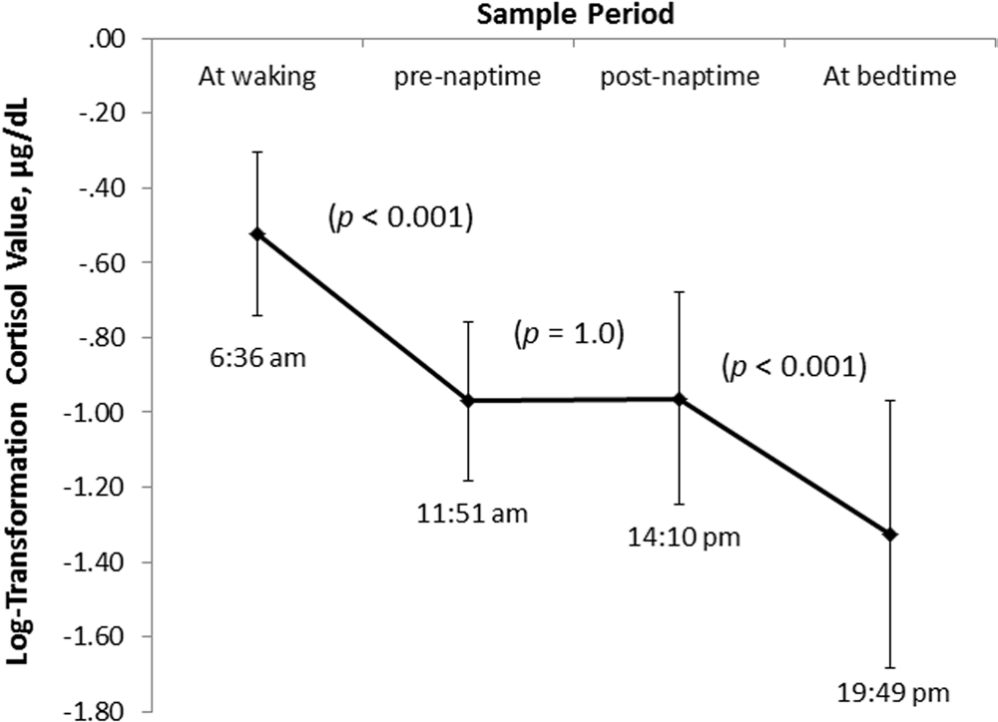
Log10 Transformed diurnal cortisol values for whole sample (*N* = 43). Error bars show standard deviation [SD]. Times shown indicate mean salivary collection time for each sample period.

Diurnal cortisol patterns are presented in Fig. 2. Significant diurnal changes in cortisol levels were detected, *F* (2.39, 100.32) = 70.98, *p* < 0.001, ${{\rm{\eta }}}_{{\rm{p}}}^{2}$ = 0.63. Post-hoc tests revealed a significant change in cortisol from wake to pre-naptime (*p* < 0.001) and from post-naptime to bedtime (*p* < 0.001). There was no significant change in cortisol between pre- and post- naptime (*p* = 1.0).

### Group Differences in Diurnal Cortisol Patterns

**Figure 3:**
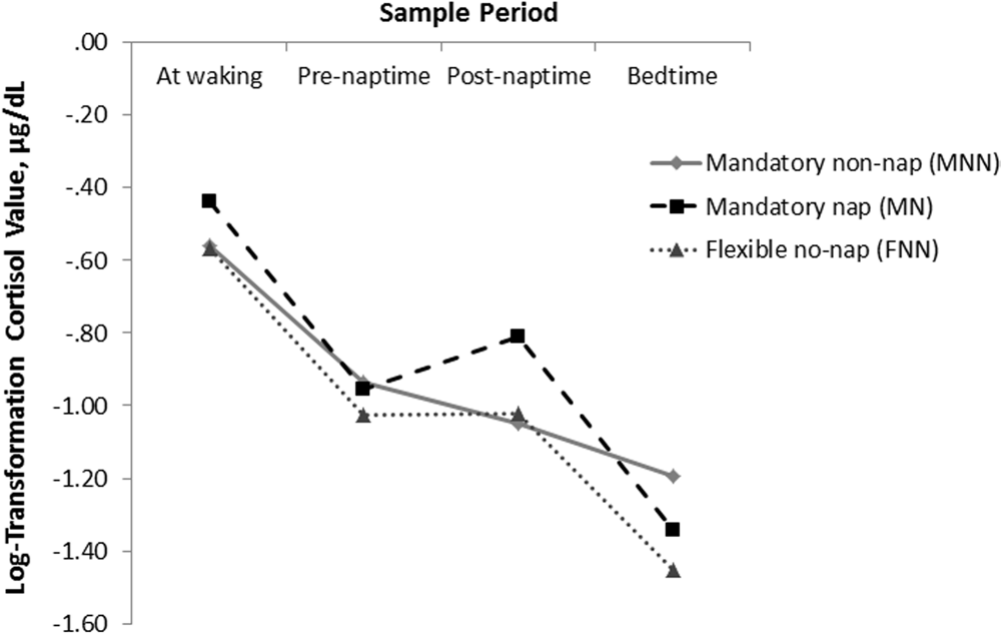
Log10 Transformed diurnal cortisol values across MNN, MN and FNN groups.

**Figure 4:**
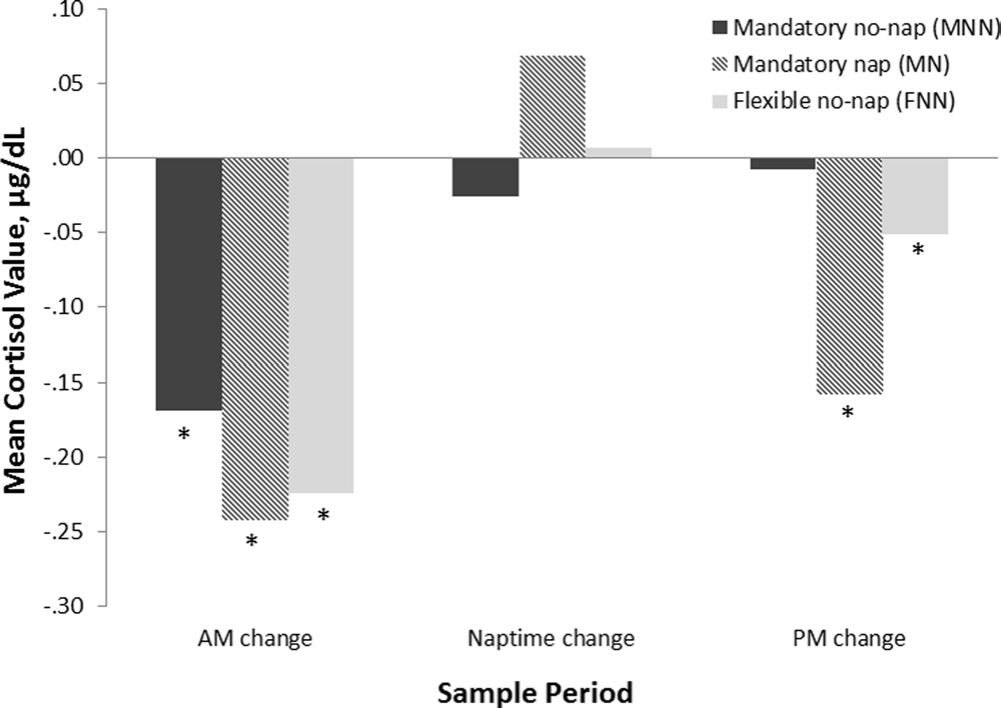
Cortisol change scores napping × naptime practice groups. ‘AM change’ = change in mean cortisol value from wake to pre-nap; ‘Naptime change’ = change in mean cortisol value from pre-nap to post-nap; ‘PM change’ = change in mean cortisol value from post-nap to bedtime. *Indicates significant within group cortisol change (*p* < 0.01).

Diurnal cortisol patterns for MNN, MN and FNN conditions are shown in Fig. 3. A significant time dependent change in diurnal cortisol patterns was identified across all study groups (MNN: *F*(1.87, 26.16) = 15.54, *p* < 0.001, ${{\rm{\eta }}}_{{\rm{p}}}^{2}$ = 0.51; MN: *F*(3, 39) = 33.32, *p* < 0.001, ${{\rm{\eta }}}_{{\rm{p}}}^{2}$ = 0.72; FNN: *F*(1.84, 23.95) = 34.30, *p* < 0.001, ${{\rm{\eta }}}_{{\rm{p}}}^{2}$ = 0.73). Across all three groups a significant decrease in cortisol from wake to pre-nap was found (FNN: *p* < 0.001; MN: *p* = 0.001; MNN: *p* = 0.002) followed by a flattening in cortisol between pre- and post-naptime periods (FNN: *p* = 1.0; MN: *p* = 0.0768; MNN *p* = 0.205). For children in FNN and MN groups there was a significant decrease in cortisol from post-naptime to bedtime (*p* = 0.003 and *p* = 0.001, respectively). However, for children within the MNN reductions in cortisol from post-naptime to bedtime were not significant (*p* = 1.0) (Fig. 4).

## Discussion

This study finds distinctive patterns of diurnal cortisol associated with the “fit” between childcare naptime practices and child sleep behaviour. Two key features in cortisol patterning across the groups are notable.

First we find no significant change in cortisol level across the naptime period, suggesting a negligible effect of naptime on stress response. For children who sleep a non-significant rise consistent with a cortisol awakening response is seen, while for non-sleeping children cortisol levels are essentially stable. This later finding is surprising given both the pervasive practice of scheduling naptime as a means of providing stress reduction^4–7^ and, observations that non-sleeping children in mandated naptimes evidence behavioural difficulty^8,15^ with associated elevated cortisol^15^. The findings do, however, align with an earlier report showing no immediate effect of sleep behaviour on cortisol patterning in childcare^12^.

Second, we find differences in patterns of decline from post-naptime at childcare through to bedtime at home. Children who napped under mandatory naptime conditions and those who did not nap under flexible conditions experienced a significant decline in cortisol level prior to bedtime, reflecting the normative diurnal decline in cortisol. In contrast those who did not nap under mandated naptime conditions did not show a significant reduction in cortisol levels. This finding raises the possibility that exposure to mandatory naptime practice for preschool aged children who do not sleep may attenuate decline in cortisol. While this could be due to the absence of a nap^15^, such a hypothesis is inconsistent with our finding that children who did not nap under flexible conditions also evidenced a significant decline. An alternative explanation for this attenuation may be that lying awake during mandatory naptimes (*M* = 102 minutes) in the absence of alternative activities may be challenging for young children and deplete their self-regulatory resources^16^. Such a mechanism could explain the attenuation of afternoon cortisol as a rebound response to high levels of effortful control during naptime^17^. This hypothesis is speculative at this point but directs attention to investigation of post-nap behavioural responses. Third, while it is common for cortisol studies to have samples of this size, caution should be taken in generalising the current findings beyond the specific context described. Finally, as our measurement was limited to four time-points, the non-significant change could reflect an unobserved intervening incline in cortisol at bedtime. Prior studies have shown an association between naptime practices and children’s nighttime sleep^18^. One mechanism that has been previously suggested^19^ is that the negative experience of mandatory naptime, for those unable to nap, becomes generalised to bedtime resistance and an attendant stress response.

There are a number of limitations that should be considered in the interpretation of the results. First, reflecting the low proportion of preschool children who nap overall, only one study child napped within the flexible naptime condition. While showing the effect of mandated naptime on sleep behaviour^7^ the absence of napping limited any conclusions about diurnal cortisol patterning of nappers under flexible naptime conditions. To achieve this aim would require much larger samples of services, or sampling of younger children where the frequency of napping is higher We have observed, however, that mandated naptimes are near universal at younger ages (1–3 years)^20^. Second, this study included cortisol measurement at 4 time-points across the day and, while allowing us to examine diurnal patterns, did not provide sufficient resolution to allow comment on patterning in the intermediary periods. Future analyses with higher sampling resolution is recommended, particularly across the period leading up to bedtime. Finally, while cortisol level was used as an index of stress it should be noted that there are individual and environmental variations, other than those that are the focus, which may affect measurement. In our methodology we have attended to control of known disruptors but acknowledge that there are inherent limitations in indexing stress via cortisol. Together, our findings indicate that childcare naptime practices and naptime behaviour have an observable effect on cortisol expression in young children. Exploration of the potential effects of the altered expression on child behaviour is needed.

## Methods

### Participants

Participating childcare rooms were drawn from a pool of 118 rooms catering for preschool aged children (3–5 years) in Brisbane, Australia, in which sleep policies were known^21^. The study design has been detailed elsewhere^22^ however, a brief outline is provided here. Rooms were randomly selected from those identified as having 2 hours of scheduled naptimes within their daily routines, with either (1) *mandatory naptime*: >1 hour in which all children must lie down without alternative activities permitted, or (2) *flexible naptime*: provision of alternative quiet activities (e.g. book reading, guided mediation) permitted for non-napping children throughout. In the original design the selection of rooms aimed to capture flexible and mandated practice in both high and low socio-economic areas. However, as none of the rooms meeting criteria for “flexible” were located in low SES areas, the sampling was limited to high SES areas only. In this way we control for social variables that may be associated with sleep need or stress. All children attending the target childcare rooms were invited to participate in the study. A total of 62 pre-school aged children (32 Males (51.6%); *M* = 56.51 months, *SD* = 5.94, and Age Range: 39.0–74.0 months) were recruited. Childcare attendance ranged between 2 and 5 days per week (*M* = 3.1, SD = 1.1). Approval for the study was obtained from the university human research ethics committee (Approval Number: 1200000046). All methods were performed in accordance with the relevant guidelines and regulations set out in this approval. Written informed consent was provided by directors, teachers and the legal guardians of each participating child. Children gave their assent to participate.

### Procedures

Families were sent a participant package containing a 14-day sleep diary, parent survey, Actiwatch 2 (MiniMitter Phillips) device, a cortisol sampling kit (4 × SalivaBio Children’s Swabs, 4 × Swab Storage Tubes [SST; pre-labelled] and detailed protocol instructions; including pictorial instructions). Within the 14-day testing period, researchers visited each participating pre-school classroom twice, on the same designated mid-week day. Researchers observed the childcare routine and environment, including sleep practices and children’s sleep behaviours and, on one visit, measured each child’s height and weight.

Salivary samples were collected on two test days from children at four time points; at waking, prior to naptime, post naptime, and bedtime. Parents collected samples at home (waking/bedtime) using the sampling kits provided while samples at childcare (pre- and post- naptime) were collected directly by the researchers. Parents were instructed to collect the first “at wake” sample as close as possible to the child’s wake time in the morning of the day of testing, prior to consumption of food or drinks. Similarly bedtime samples were collected immediately prior to bedtime, and not within 30 minutes of food or drink consumption. Pre-naptime cortisol samples were collected following the morning play session and prior to lunch time in childcare. Lunch times directly proceeded the naptime period. Post-naptime samples were collected immediately following the naptime, defined as the time at which children were permitted to leave their beds. For children who napped, the average time between children waking from naps and cortisol testing was 28.3 minutes (*SD* = 16.7 minutes). Parents completed a diary on each testing day recording food intake, medication use, illness, and activity levels prior to data collection points. Equivalent items were completed via direct observation by research staff whilst at the childcare service.

### Measures

#### Salivary Cortisol

Saliva was collected following the Salimetrics collection protocol, using SalivaBio Children’s Swabs (Salimetrics), designed for use in children aged between 6 months and 6 years^23^. One end of the swab was held by the researcher/parent while the other end was placed under the child’s tongue for between 60 and 90 seconds, until the swab was saturated. To support the child’s adherence to the saliva collection duration in both the childcare and home contexts, a coloured 30 second sand-timer was provided for the child to observe and turn at 30 seconds intervals until collection was complete. The swab was then placed into a labelled SST and refrigerated. Returned samples were stored in a temperature controlled freezer at below −20 °C at the research institute prior to laboratory analysis. Samples were sent to an independent laboratory (Stratech Scientific APAC Pty Ltd) for analysis. Cortisol biomarker tests were conducted in duplicate.

#### Naptime Practices

Observation of napping practices utilised the Sleep Observation Measure for ECEC^8,24^. Consistent with the SOME^8,24^, mandatory naptime was coded in minutes, and reflected the length of time during which all children were required to lie on beds, without any alternate activity permitted. Prior studies indicate high inter-rater reliability (intra-class correlation 0.996) in determining sleep practices using this method^8,24^.

#### Napping Behaviours

Children wore an Actigraph for 14-days on their non-dominant wrist. Actigraphy measured children’s motor activity (range: 0.5–2 G) in 1 minute epochs. Rest/sleep intervals were assessed using parent reported sleep diaries and wrist actigraphy, using Actiware 5.2 software (Phillips Respironics, Bend, Oregon 97701 USA). The criteria for determining sleep patterns has been published previously^22^. Where actigraphy data were unavailable (*N* = 12 of the original 62 participants), observational data from the testing days were substituted using the SOME napping observation protocol^24^. This method requires trained observers to code child behaviours into five categories (asleep, potentially asleep, awake, away from bed, disruptive/distressed) at 10-minute intervals. Consistent with prior studies^7^ excellent reliability of observations and ambulatory devices was found ICC > 0.94.

#### Control Variables

Height and weight were measured by trained researchers using standardised protocols^22,25^. BMI measurements were standardised into sex- and age-specific *z*-scores using the World Health Organisation’s Anthro version 3.2.2 and AnthroPlus version 1.0.4. Family demographics, including income and parent education were derived from parent questionnaires. Parents also completed the Short Temperament Scale for Children^26^ and the Strengths and Difficulties Questionnaire^27^.

### Data analysis

A number of exclusion criteria were applied in deriving the final sample. From the total saliva collection, 10 (2.6%) samples had insufficient volume to allow analysis. To control for potential contamination of samples, samples yielding biologically implausible values were excluded by applying a criteria >3 SD above the mean for each time point. As such, a further 7 samples (1.8%) were excluded. Parent report diaries and observation data were also examined for events that might contaminate samples. Ten [2.6%]) samples were excluded due to consumption of food and/or milk products within 30 minutes prior to sample collection. No children were noted as undertaking physical activity within the hour prior to sample collection. Medication use was examined and cross-checked with a pharmacist to identify presence of steroidal types. No steroidal medication use was identified within the sample. One child was identified as having a diagnosed developmental disorder and was removed from all analyses. In addition, 4 children were reported by parents as being diagnosed with asthma, however as these children were not taking Salbutamol medication at the time of data collection these children were retained in all subsequent analyses.

In line with the methods used by Watamura *et al*.^13^ absolute cortisol levels from each time point (at waking, prior to naptime, post naptime, and bedtime) were averaged across the two testing days to provide an average cortisol score. Where children had samples for one day and not the other (2 children), the scores for this day only were included in analyses. Three children were identified as napping on one day of data collection and not the other. These three children were subsequently randomly allocated to napping groups with corresponding cortisol data included in analyses. This provided a total sample of 43 children for analysis.

Analyses were conducted using SPSS Version 23. To account for potential confounding variables that may influence cortisol levels, group differences for child age, gender, time of cortisol sampling, child temperament, family income, parent education and BMI-z scores were initially examined (Table 2). Tests of group differences were conducted using independent groups ANOVA with post-hoc tests with Bonferonni adjustment and Pearson’s Chi-Squared tests. Where breaches to normality were identifies, non-parametric analyses were applied.

Diurnal cortisol patterns were examined via repeated measures ANOVA of cortisol levels across 4 time points (wake, pre-naptime, post-naptime and bedtime). To account for a positive skew in cortisol data Log10 transformations were applied to mean cortisol values prior to analyses. Due to the sample size, diurnal cortisol patterns were examined separately for each of the FMM, MNN and MN conditions. Where breaches to sphericity assumptions were detected, Greenhouse-Geisser correction was applied. Post-hoc tests with Bonferroni adjustment were performed.

### Data availability statement

The datasets generated and analysed during the current study are available from the corresponding author on reasonable request.

## Electronic supplementary material

Supplementary Table 1
