# Supplementary material for: Mandatory Naptimes in Childcare do not Reduce Children’s Cortisol Levels
Source: Sci Rep. 2018 Mar 14;8:4545. doi: 10.1038/s41598-018-22555-8 (PMC5852241; doi:10.1038/s41598-018-22555-8)
Supplement: Supplementary file 1 — Supplementary Table 1 [file 41598_2018_22555_MOESM1_ESM.docx]

**Supplementary Information**

**Title:** Mandatory Naptimes in Childcare do not Reduce Children’s Cortisol Levels

**Authors:** Karen J. Thorpe^1*^, Cassandra L. Pattinson^1^, Simon S. Smith^1^, Sally L. Staton^1^.

**Affiliations:**

^1^ Institute for Social Science Research (ISSR), The University of Queensland, Long Pocket Precinct, 80 Meiers Rd, Indooroopilly, Queensland, Australia, 4068

*Correspondence to: k.thorpe@uq.edu.au

**Table 1.** Correlation coefficients for mean cortisol levels by covariates.

|  | | | | | |
| --- | --- | --- | --- | --- | --- |
|  | Correlation Coefficient^†^  Mean Cortisol level | | | | |
|  |  |  |  |  |  |
|  |  | Wake | Pre-naptime | Post-naptime | Bedtime |
| Age [months] |  | -.12 | -.19 | .14 | -.16 |
| Sampling time |  |  |  |  |  |
| Wake |  | -.09 | -.04 | .27 | .13 |
| Pre-nap |  | .02 | -.12 | -.14 | -.02 |
| Post-nap |  | .03 | -.26 | -.23 | .01 |
| Bedtime |  | .10 | .17 | .20 | -.30 |
| BMI-Z score |  | .14 | .14 | .14 | .23 |
| Child temperament |  | .01 | -.16 | -.23 | .08 |
| Total Difficulties Score |  | -.07 | .04 | .18 | -.25 |
| Family income |  | -.03 | .11 | .07 | .21 |
| Parent education |  | -.06 | .12 | -.33***** | -.11 |
| *Note.* MNN= mandatory no-nap; MN = mandatory nap; FNN = flexible no-nap.^†^Spearman’s rho. ******p*=.04. | | | | | |
